# The diagnostic value of the bronchoalveolar lavage in interstitial lung diseases

**DOI:** 10.1186/s12952-017-0069-0

**Published:** 2017-03-01

**Authors:** Boubacar Efared, G. Ebang-Atsame, Sani Rabiou, Abdoulsalam S. Diarra, Layla Tahiri, Nawal Hammas, Mohamed Smahi, Bouchra Amara, Mohamed C. Benjelloun, Mounia Serraj, Laila Chbani, Hinde El Fatemi

**Affiliations:** 1 Department of Pathology, Hassan II Teaching Hospital, (B.P. 1835, Atlas) Route de sidi Harazem Fès - Maroc, Fez, Morocco; 2 Department of Thoracic Surgery, Hassan II Teaching Hospital, Fez, Morocco; 3 Department of Epidemiology, Faculty of Medicine and Pharmacology, Fez, Morocco; 4 Faculty of Medicine and Pharmacology, Fez, Morocco; 5 Department of Pneumology, Hassan II Teaching Hospital, Fez, Morocco

**Keywords:** Bronchoalveolar lavage, Interstitial lung diseases, Diagnostic, Cytology

## Abstract

**Objective:**

Bronchoalveolar lavage (BAL) is a diagnostic tool often used during the management of interstitial lung diseases (ILD). However, its diagnostic value in discrimination between entities comprising the very heterogenous group of ILD, is still a controversial issue. The objective of our study is to assess the diagnostic value of BAL in the management of ILD, by comparing the cytological findings in BAL fluid among the different diseases of this group.

**Methods:**

It was a retrospective, observational study of 151 patients between January 2012 and December 2015. BAL fluid cytology was performed to analyse the distribution of leucocytes population subsets in patients with ILD.

**Results:**

The mean age was 52.78 years; 74.83% were women. The analysis of the following main groups of diseases was performed : sarcoïdosis (*n* = 30), idiopathic pulmonary fibrosis (IPF; *n* = 22), other idiopathic interstitial pneumonia (non specific interstitial pneumonia, cryptogenic organising pneumonia and respiratory bronchiolitis interstitial lung disease; *n* = 20) and connective tissue disease (*n* = 14).

Overall, out of 141 patients, 22% had sarcoïdosis, 15.6% had idiopathic pulmonary fibrosis (IPF), 14.18% had other idiopathic interstitial pneumonia (IIP) and 9.9% had connective tissue disease (CTD). Mixed alveolitis was common in the 4 groups, sarcoïdosis had higher proportion of lymphocytes and IPF had higher neutrophils count. However, there was no significant statistical difference of BAL cellular count among these diseases (*p > 0.05*). Also, the prevalence of studied diseases did not change with variation of BAL cellular count (*p > 0.05*).

**Conclusion:**

Alone, the BAL cytological analysis has a limited value to provide substantial information that could lead to discriminate between diseases that form ILD. Thus, it must be always associated with other diagnostic methods.

## Introduction

BAL is a non-invasive procedure performed with the fiberoptic bronchoscope in a wedge position within the selected bronchopulmonary segment. The examination of cells and solutes from the lower respiratory tract provides valuable information about diagnosis and yield insights into immunologic, inflammatory, and infectious processes taking place at the alveolar level [1–3]. The cytological analysis of BAL fluid is commonly used in the management of a variety of lung diseases especially the large and wide group of interstial lung diseases (ILD) [1, 4, 5]. The term of ILD consisted of acute and chronic bilateral parenchymal infiltrative lung diseases with variable degrees of tissue inflammation and fibrosis when they occur in immunocompetent hosts without infection or neoplasm [1]. ILD can be either of known or unknown cause; according to the statement of the American Thoracic Society and the European Respiratory Society, ILD with known cause include the pneumoconioses, ILD associated with connective tissue disease (CTD-ILD), and hypersensitivity pneumonitis (HP); ILD with unknown cause are sarcoidosis and idiopathic interstitial pneumonias (IIP) [1]. IIP are another heterogenous entity comprising idiopathic pulmonary fibrosis (IPF), nonspecific interstitial pneumonia (NSIP), desquamative interstitial pneumonia (DIP), respiratory bronchiolitis with interstitial lung disease (RBILD), acute interstitial pneumonia (AIP), cryptogenic organizing pneumonia (COP), and lymphoid interstitial pneumonia (LIP) [1–4].

The diagnosis of ILD relies on a combination of multiple diagnostic tools, such as imaging technics (especially high resolution computed tomography (HRCT), blood test, lung function tests, transbronchial biopsy or lung biopsy [1, 3, 4]. All these diagnostic modalities should be correlated to a clinical context of the patient: physical examination, detailed clinical history, smoking history,…etc. Transbronchial biopsy is very helpful in the diagnosis of malignancy or granulomatous diseases but lacks any specificity in ILD. The lung biopsy is an invasive technics that can be performed via thoracoscopy or thoracotomy, but often it can be contra-indicated in some patients because it is associated with morbidity and mortality [1, 3]. The diagnostic value of BAL cytological analysis in the management of ILD is still a matter of debate and controversis [1, 4, 6, 7]. Thus, the objective of our study is to analyse BAL cytological findings in the most common ILD in order to assess its diagnostic value in the differential diagnosis of these diseases.

## Methods

### Patients

Over a period of 4 years (January 2012-December 2015), we included retrospectively, 151 cases of BAL in patients suspected of ILD, registered in the service of anatomical and cytological pathology of Hassan II teaching hospital, Fez Morocco. The diagnosis of ILD has been based on confrontations of clinical, biological and cyto-histological aspects, according to international consensus [1, 8]. All cases of ILD have been discussed in multidisciplinary meetings attended by diverse specialists: pathologists, pneumologists, oncologists, radiologists, radio-oncologists and thoracic surgeons.

### BAL cellular analysis

Collected BAL fluids have been cytocentrifuged and stained with Wright-Giemsa stain, Perls stain, and PAS stain for total and differential cell counts. BAL cytological analysis has been performed manually by a pathologist specialised in cytology. Differential cell count has been performed with identification of alveolar macrophages, lymphocytes, neutrophils and eosinophils, or other findings like tumoral cells, foreign body, mastocytes, basophils or red blood cells.

The analysis and comparison of differential cell count were carried out among the common ILD or group of ILD encountered : sarcoidosis, connective tissue diseases (CTD), idiopathic pulmonary fibrosis (IPF), nonspecific interstitial pneumonia (NSIP), cryptogenic organizing pneumonia (COP) and respiratory bronchiolitis with interstitial lung disease (RBILD). NSIP, COP and RBILD have been associated in a single groupe of other IIP, because of their small prevalence in our study, and because of their prognostic similarities, compared to IPF.

### Statistical analysis

In the descriptive analysis, qualitative variables were expressed as absolute and relative frequencies and quantitative variables as means and standard deviations. Differences in BAL cytology with macrophages, lymphocytes and polymorphonuclear cells subsets among groups were compared by using the one-way ANOVA tables. Pearson’s Chi-square test was performed to assess the prevalence changes of different ILD diseases according to differential cell count variations. The variation of inflammatory cell subpopulations has been arbitrarely fixed as intervals, according to normal values and in regard to previous studies [1, 5, 9, 10].

All statistical analyses were performed by using SPSS 20.0 version software for Windows (SPSS, Inc., Chicago, IL, USA). P value was considered statistically significant at *P* < 0.05.

## Results

Our study included 151 patients, with 113 females and 38 males (sex ratio Male/female = 0.33). The mean age was 52.78 years (age variying from 15 to 80 years).

Of 151 patients, 141 had definitive diagnosis (Table 1). Sarcoidosis was the most encountered disease, followed by IPF. IIP (IPF, NSIP, POC and RBILD) were found 42 patients, with a prevalence of 29.79%, IPF was the most diagnosed IIP.

**Table 1:** Final diagnosis

Diseases	No. (%)
Sarcoidosis	30 (22)
IPF	22 (15.6)
CTD	14 (9.9)
RBILD	10 (7.1)
EAA	10 (7.1)
Vasculitis	9 (6.4)
Tuberculosis	9 (6.4)
Heart diseases	7 (5)
NSIP	6 (4.3)
Pneumoconiosis	6 (4.3)
COP	4 (2.8)
Tumors	4 (2.8)
Eosinophilic pneumonia	4 (2.8)
Drug-induced pneumonitis	3 (2.1)
Infectious pneumonitis	3 (2.1)
Total	141 (100)

*IPF* idiopathic pulmonary fibrosis, *CTD* Connective tissue diseases, *RBILD* respiratory bronchiolitis-associated interstitial lung disease, *EAA* extrinsic allergic alveolitis, *NSIP* nonspecific interstitial pneumonia, *COP* cryptogenic organizing pneumonia

Table 2 shows the cell differentials according to final diagnoses in the main groups with ILD (sarcoidosis, IPF, CTD and other IIP (NSIP, POC and RBILD). These diseases showed mixed alveolitis (lymphocytes and neutrophils), sarcoidosis had the highest lymphocytes count (38.13%) followed by CTD (29.07%). Mixed alveolitis with predominant neutrophils count was observed in IPF (18.23% of neutrophils). However these differences between studied disesases were not statistically significant as p˃0.05 in all cellular counts.

**Table 2:** BAL cellular count in studied interstitial lung diseases (ILD)

	Sarcoidosis (*n* = 30)	IPF (*n* = 22)	Other IIP (*n* = 20)	CTD (*n* = 14)	*P value*
Macrophages (%)	46.1 (22.87)	55.5 (23.93)	56.64 (20.62)	52.27 (32.34)	0.4
Lymphocytes (%)	38.13 (26)	26.7 (19.23)	29.07 (22.15)	30.71 (32.18)	0.23
Neutrophils (%)	14.22 (18.13)	14.97 (23.65)	13 (12.7)	15.21 (14.79)	0.82
Eosinophils (%)	1.89 (5.24)	2.39 (1.27)	1.15 (3.86)	1.57 (1.4)	0.48

Data are presented as means (standard deviation). *IIP* idiopathic interstitial diseases. Other IIP includes NSIP, COP and RBILD

Few patients (37 cases) in our study had immunophenotyping of CD4 and CD8 lymphocytes (Table 3). The overall mean of CD4/CD8 ratio was 2.18. The mean CD4/CD8 ratio was highest for sarcoidosis compared to other diseases, it was 2.56.

**Table 3:** CD4/CD8 ratio

CD4/CD8			
	Mean	˂3.5	≥3.5
Sarcoïdosis (*n* = 13)	2.65	9	4
IPF (*n* = 4)	1.5	3	1
Tuberculosis (*n* = 4)	1.99	4	0
EAA (*n* = 5)	1.41	5	0
RBILD (*n* = 2)	2.19	1	1
Pneumoconiosis (*n* = 2)	1.66	2	0
POC (*n* = 1)	1.7	1	0
CTD (*n* = 1)	1.34	1	0
Vasculitis (*n* = 1)	1.6	1	0
Eosinophilic pneumonitis (*n* = 1)	6.25	0	1
Heart diseases (*n* = 1)	2.8	1	0
Tumors (*n* = 1)	1.4	1	0
Infectious pneumonitis (*n* = 1)	2.35	1	0

The Pearson’s Chi-square test has been performed to assess the prevalence changes of studied ILD, when BAL differential count of cellular subpopulations varies in certain proportions.

The Table 4 shows that the prevalence of sarcoidosis was high when lymphocytes > 40%. The prevalence of IPF and other IIP decreased when lymphocytes count increased. However these changes in prevalence according to lymphocytes count variation were not statistically significant (*p* = 0.33).

**Table 4:** Prevalence of studied ILD according to lymphocytes count variation

Lymphocytes (%)			
	≤20	21–40	> 40
Sarcoïdosis (*n* = 30)	36.7%	20%	43.3%
IPF (*n* = 22)	45.5%	36.4%	18.2%
Other IIP (*n* = 20)	50%	30%	20%
CTD (*n* = 14)	57.1%	14.3%	28.6%

Prevalence variation of studied ILD is not statistically significant (*p* = 0.33)

Also, the prevalence according to neutrophils count variation was not statistically significant (*p* = 0.38), as illustrated in Table 5. The prevalence of sarcoidosis decreased when neutrophils count > 20%. The prevalence of IPF was maximal when neutrophils range between 5 and 20%. CTD were frequently diagnosed when neutrophils count < 5%.

**Table 5:** Prevalence of studied ILD according to neutrophils count variation

Neutrophils (%)			
	< 5	5–20	> 20
Sarcoïdosis (*n* = 30)	40%	40%	20%
IPF (*n* = 22)	27.3%	45.5%	27.3%
Other IIP (*n* = 20)	20%	60%	20%
CTD (*n* = 14)	50%	21.4%	28.6%

Prevalence variation of studied ILD is not statistically significant (*p* = 0.38)

Similarly, the eosinophils count did not affect the prevalence of studied ILD diseases (Table 6), (*p* = 0.05). IPF was more freqently diagnosed when neutrophils range between 2 and 5%, the prevalence of other diseases decrased when neutrophils count > 1%.

**Table 6:** Prevalence of studied ILD according to eosinophils count variation

Eosinophils (%)			
	≤1	2–5	> 5
Sarcoïdosis (*n* = 30)	80.8%	11.5%	7.7%
IPF (*n* = 22)	27.3%	85.7%	0.0%
Other IIP (*n* = 20)	47.4%	47.4%	5.3%
CTD (*n* = 14)	61.5%	38.5%	0%

Prevalence variation of studied ILD is not statistically significant (*p* = 0.05)

Table 7 showed that the prevalence of all studied ILD increased when macrophages count augments, but these prevalence changes were not statistically significant (*p* = 0.35).

**Table 7:** Prevalence of studied ILD according to macrophages count variation

Macrophages (%)			
	< 30	30–50	> 50
Sarcoïdosis (*n* = 30)	26.7%	33.3%	40%
IPF (*n* = 22)	13.6%	27.3%	59.1%
Other IIP (*n* = 20)	5%	35%	60%
CTD (*n* = 14)	21.4%	14.3%	64.3%

Prevalence variation of studied ILD is not statistically significant (*p* = 0.35)

## Discussion

In the current study, we have tried to assess the diagnostic value of BAL in ILD by analysing and comparing the different cellular subpopulations count between the most commonly diagnosed ILD, such as sarcoidosis, CTD and certain IIP (IPF, NSIP, POC and RBILD). The diagnosis of these diseases is not easy; thus it relies on a confrontation between clinical, biological, radiological, histological and cylogical features of the patient [1, 7, 8]. ILD are a very heterogenous group of diseases including a variety of clinical entities that do not share the same prognosis [1–5]. Also, the treatment varies among diseases that form the large group of ILD, hence the urge of exact etiological diagnosis in order to ajust treatment according to the causative disease [1, 7–13]. Unfortunately, all available diagnostic tools (radiology, biology, cytology, histology) lack any specificity [1, 2, 6, 14–16]. In our study, we found that all studied ILD were characterised by mixed alveolitis with predominant lymphocytes and neutrophils count, sarcoidosis had the highest lymphocytes count (38.13%). But statistical analysis failed to show any significant difference between studied diseases. We have also tried to know if the prevalence of studied ILD changes with the variation of different subpopulations count (lymphocytes, macrophages, neutrophils and eosinophils). However, we found that the variation in prevalence of ILD was not significantly affected by BAL differential count variation. For instance, when lymphocytes count > 40%, the prevalence of sarcoidosis was high, when this count ≤ 20%, the prevalence of CTD, IPF, and other IIP, increased. When it comes to neutrophils count, the prevalence of sarcoidosis decreased when neutrophils count > 20%, the prevalence of IPF was high when neutrophils range between 5 and 20%, CTD were mostly diagnosed when neutrophils count was lower than 5%. As mentionned above, all these findings were not statistically significant. In the literature, it was widely reported that sarcoidosis shows high lymphocytes count associated with CD4/CD8 > 2 [9, 16–18]. In our study, few patients (37 cases) had lymphocytes immunophenotyping. The mean CD4/CD8 was 2.56 for sarcoidosis. We have also found a high neutrophils count for IPF, as reported in the literature [2, 9]. In the presence of a high neutrophils count associated with a mild to moderate lymphocytes count (< 30% in general), the diagnosis of IPF should be considered [2, 3, 9].

The diagnostic value of BAL to discriminate between ILD is still a challenging and controversial issue. Numerous studies have tried to deal with this issue, leading to contradictory conclusions, and some authors claimed the limited clinical usefulness of BAL cellular analysis in ILD [6]. Welker L. et al. showed that the likelihood for sarcoidosis increased from 33.7 to 68.1% when lymphocyte numbers were 30–50% and granulocyte numbers were low; the likelihood for usual interstitial pneumonia (UIP) increased from 15.8 to 33.3% when lymphocyte numbers were < 30% with granulocytes elevated [9]. This study, like many other studies pointed out the diagnostic value of CD4/CD8 in the diagnosis of sarcoidosis [6, 16–18]. Eosinophils are very rare in BAL fluid, they range between 0 and 1%, any eosinophils count higher than this range is pathological [1, 3, 5]. BAL eosinophilia can be found at variable degrees in diseases like immuno-allergic pneumonia or chronic eosinophilic pneumonia; also mild to moderate eosinophilia can be found in ILD [1, 7, 11]. We found that IPF was associated with a mild hypereosinophilia (2.39%), in the literature it has been reported that BAL eosinophilia associated with a mild lymphocytes count and elevated neutrophils, is an important feature of IPF [4, 8, 9]. BAL in CTD has no particular characteristics, often mixed alveolitis with predominant lymphocytes and neutrophils, was found [9, 19].

Despite limitations (small number of patients, retrospective and monocentric study), the findings in our study prompted us to question the real utility of BAL cellular count in ILD. In fact, we found any statistical difference in BAL cytology between studied ILD diseases. Recently, a study by Lee W et al. on 69 cases of ILD concluded that the routine analysis of BAL lymphocyte subset may not provide any additional benefit for differential diagnosis of DILDs, except for conditions where BAL is specifically indicated, such as eosinophilic pneumonia or sarcoidosis [6]. It has been speculated that BAL plays an important role in the management and follow-up of patients treated for ILD [1, 3, 10], however another recent study by Petrosyan F et al. on 77 patients followed for IPF, showed that pulmonary infection can be excluded based on clinical and laboratory data and that bronchoscopy with BAL is not mandatory in the diagnostic work-up of suspected acute clinical deterioration of IPF [20].

Despite tremendous controversis, it is widely accepted that BAL analysis alone has no diagnostic value, confrontation with clinical and radiological features is necessary [1]. That is why in our center, all cases of ILD are discussed in multidisciplinary meetings (MDM), as it is widely accepted that these meetings are very useful in the management of ILD [21, 22]. The American Thoracic Society (ATS) statement has been clear and realistic. “When used in conjunction with comprehensive clinical information and adequate thoracic imaging such as high-resolution computed tomography of the thorax, BAL cell patterns and other characteristics frequently provide useful information for the diagnostic evaluation of patients with suspected ILD” [1].

## Conclusion

We found in the current study that there was no statistically significant difference in BAL cytology between ILD. Thus BAL doesn’t provide any substantial information that could lead to discriminate between entities that form ILD. The definitive diagnosis of ILD should be discused in MDM after confrontation between BAL cytology and other dignostic tools.

## Abbreviations

BAL: Bronchoalveolar Lavage
COP: Cryptogenic organizing pneumonia
CTD: Connective tissue diseases
EAA: Extrinsic allergic alveolitis
IIP: Idiopathic interstitial diseases.
ILD: Interstitial lung diseases
IPF: Idiopathic pulmonary fibrosis
MDM: Multidisciplinary meeting
NSIP: Nonspecific interstitial pneumonia
RBILD: Respiratory bronchiolitis-associated interstitial lung disease
